# Diversity, Distribution, and Chromosomal Rearrangements of TRIP1 Repeat Sequences in *Escherichia coli*

**DOI:** 10.3390/genes15020236

**Published:** 2024-02-13

**Authors:** Zhan Li, Xiong Liu, Nianzhi Ning, Tao Li, Hui Wang

**Affiliations:** 1 State Key Laboratory of Pathogens and Biosecurity, Beijing Institute of Microbiology and Epidemiology, No. 20 Dongda Street, Fengtai District, Beijing 100071, China; yexi19881214@126.com (Z.L.); ningnianzhi@163.com (N.N.); litaobmi@126.com (T.L.); 2 Chinese PLA Center for Disease Control and Prevention, Dongda Street 20#, Fengtai District, Beijing 100071, China; liuxiong714@163.com

**Keywords:** TRIP1 repeats, *Escherichia coli*, chromosomal rearrangement

## Abstract

The bacterial genome contains numerous repeated sequences that greatly affect its genomic plasticity. The *Escherichia coli* K-12 genome contains three copies of the TRIP1 repeat sequence (TRIP1a, TRIP1b, and TRIP1c). However, the diversity, distribution, and role of the TRIP1 repeat sequence in the *E. coli* genome are still unclear. In this study, after screening 6725 *E. coli* genomes, the TRIP1 repeat was found in the majority of *E. coli* strains (96%: 6454/6725). The copy number and direction of the TRIP1 repeat sequence varied in each genome. Overall, 2449 genomes (36%: 2449/6725) had three copies of TRIP1 (TRIP1a, TRIP1b, and TRIP1c), which is the same as *E. coli* K-12. Five types of TRIP1 repeats, including two new types (TRIP1d and TRIP1e), are identified in *E. coli* genomes, located in 4703, 3529, 5741, 1565, and 232 genomes, respectively. Each type of TRIP1 repeat is localized to a specific locus on the chromosome. TRIP1 repeats can cause intra-chromosomal rearrangements. A total of 156 rearrangement events were identified, of which 88% (137/156) were between TRIP1a and TRIP1c. These findings have important implications for future research on TRIP1 repeats.

## 1. Introduction

DNA repeats have been defined as sequences with high similarity to other sequences found in the same genome, which were first identified in a bacterial genome during the early 1980s [1]. It has been discovered that repeat sequences are a common feature of bacterial genomes. Bacterial genomes contain numerous repeat sequences that can be classified based on several criteria [2]. These include their direction (direct or inverted), size (simple short or long), distribution (contiguous or interspersed), and copy number with the genome. The large number of repeat sequences found in the bacterial genome has attracted much attention regarding their origin, distribution [3], rearrangement [4], and influence on the bacterial genome [5].

DNA repeat sequences have divergent functions in complex DNA processes, such as replication, recombination, repair, and transcription [6]. Through their involvement in these processes, DNA repeat sequences influence the genetic instability and evolution of DNA molecules [7]. DNA repeat sequences that are rich in GCs, as triple hydrogen bonds, could stabilize DNA structures to facilitate bacterial survival [8]. DNA repeats are inserted into the transcription units of surrounding sequences, affecting their transcription and translation [9]. The removal of these DNA sequences led to improper folding and instability of the protein structure [10]. Therefore, DNA repeat sequences play a critical role in an organism’s physiology and cellular architecture.

DNA repeats vary in length from dinucleotides to thousands of nucleotides and are widespread in bacterial genomes. DNA repeats of the length of at least 100 nucleotides are called long DNA repeats [11]. Long identical DNA repeats are often distributed in a non-random manner across bacterial genomes. Their distribution differs in the overall number, orientation, and proximity to the origin of replication. In addition to some functional DNA repeat sequences [12,13,14], many long DNA repeats have been found to be closely related to the structural evolution of bacterial genomes [15,16,17,18]. Long DNA repeats are major players in mediating rearrangements. Long DNA repeats can lead to intrachromosomal rearrangements by acting as substrates to the bacterial recombination machinery. Rearrangement results were found to depend on the location and direction of the associated repeats, which change the order of genes on the chromosome [15,18,19]. Certain mobile elements have also targeted DNA repeat sequences [20,21,22]. DNA repeat analysis in bacterial genomes can provide valuable information on genomic plasticity among microbial species; moreover, changes in genome structure affect function and biological processes [23,24,25]. The analysis can reveal important insights into the genetic makeup of these organisms.

TRIP1 repeat sequences are 146 to 273 bp in length, and three copies (TRIP1a, TRIP1b, and TRIP1c) of TRIP1 repeat sequences are found in the complete *Escherichia coli* K-12 strain genome sequence [26]. The three copies are all located at different chromosomal locations. TRIP1a is 31 bp downstream of the apparent aqpZ transcription terminator. TRIP1b is 41 bp downstream of the putP stop codon and is a partial form of TRIP1a, with 125 bp internal deletion. TRIPc is 44 bp downstream of the yohI gene, in an opposite orientation relative to TRIP1a. The copy number, distribution, and influence of the TRIP1 repeat sequence on the *E. coli* genome are not very clear. In this study, a total of 6725 *E. coli* genomes were scanned for the presence of the TRIP1 repeat sequence, and the types, distribution, and influence of TRIP1 on the *E. coli* genome were investigated.

## 2. Materials and Methods

### 2.1. Genome Sequences

*E. coli* genome sequences were downloaded from the NCBI ftp site (https://ftp.ncbi.nlm.nih.gov/genomes/refseq/bacteria/ accessed on 8 October 2016). In total, 6725 *E. coli* genome sequences were available for analysis and were downloaded using Wget V1.18 software (https://eternallybored.org/misc/wget/ accessed on 8 October 2016).

### 2.2. Identification and Localization of the TRIP1 Repeat Sequences

The query sequence for the BLAST search consisted of all three copies of the TRIP1 (TRIP1a, TRIP1b, and TRIP1c) repeat sequence found in *E. coli* K-12 (NC_000913.3). TRIP1a was located on the top strand from 915016 to 915281, TRIP1b from 1080855 to 1081000, and TRIP1c from 2229122 to 2229394. The gap opening cost was set to 5, and gap the extension was set to 2. 

The BLAST search results were analyzed as follows. First, all matching sequences with length coverage greater than 80% were compared and clustered using USEARCH manual v8.1 software (available at https://drive5.com/usearch/manual8.1/ accessed on 25 August 2017) [27]. Two previously undiscovered TRIP1 types (TRIP1d and TRIP1e) were identified in the chromosomes of other *E. coli* strains. TRIP1d was located on the top strand from 2251149 to 2251412 in *E. coli* 2012C-4502 (CP027440.1) and TRIP1e from 1232542 to 1232806 in *E. coli* RHB34-C04 (CP057178.1).

The five types of TRIP1 repeat sequences were compared and aligned using BioEdit 7.7 [28] and ClustalW 7.7 [29] software. Second, only the highest-scoring TRIP1 type was used for subsequent analysis when multiple types of TRIP1 repeat sequences were matched at the same position in a genome. Third, to determine the position of the TRIP1 repeat sequence in each genome, the 1000 bp DNA sequences flanking both sides were extracted and annotated using the KEGG gene database (https://www.genome.jp/kegg/ accessed on 17 October 2023). 

### 2.3. RNA Structure Prediction of the TRIP1 Repeat Sequences

The RNAfold WebServer (http://rna.tbi.univie.ac.at/cgi-bin/RNAWeb-Suite/RNAfold.cgi/ accessed on 27 October 2023) was used to depict the most stable folded RNAs hypothetically expressed by the TRIP1a, TRIP1b, TRIP1c, TRIP1d, or TRIP1e repeat sequences.

### 2.4. Identification of Chromosomal Arrangement Caused by TRIP1 Repeat Sequences

To determine the chromosomal arrangement resulting from the TRIP1 repeat sequences, all genome sequences were analyzed as follows. First, for each of the identified TRIP1 loci, the two flanking genes were searched in the entire genome sequence. If an arrangement event occurred between two of the loci, the relative position and distance of the four associated genes would change, generating a new permutation between the flanking genes. All genomes with novel permutations between the locus-flanking genes were recorded. Second, the rearrangement events between the complete genome sequences were analyzed using Mauve 2.3.0 software (https://darlinglab.org/mauve/mauve/ accessed on 1 November 2023). 

## 3. Results 

### 3.1. The Distribution and Copy Number of TRIP1 Repeats in E. coli Genomes

**Figure 1:**
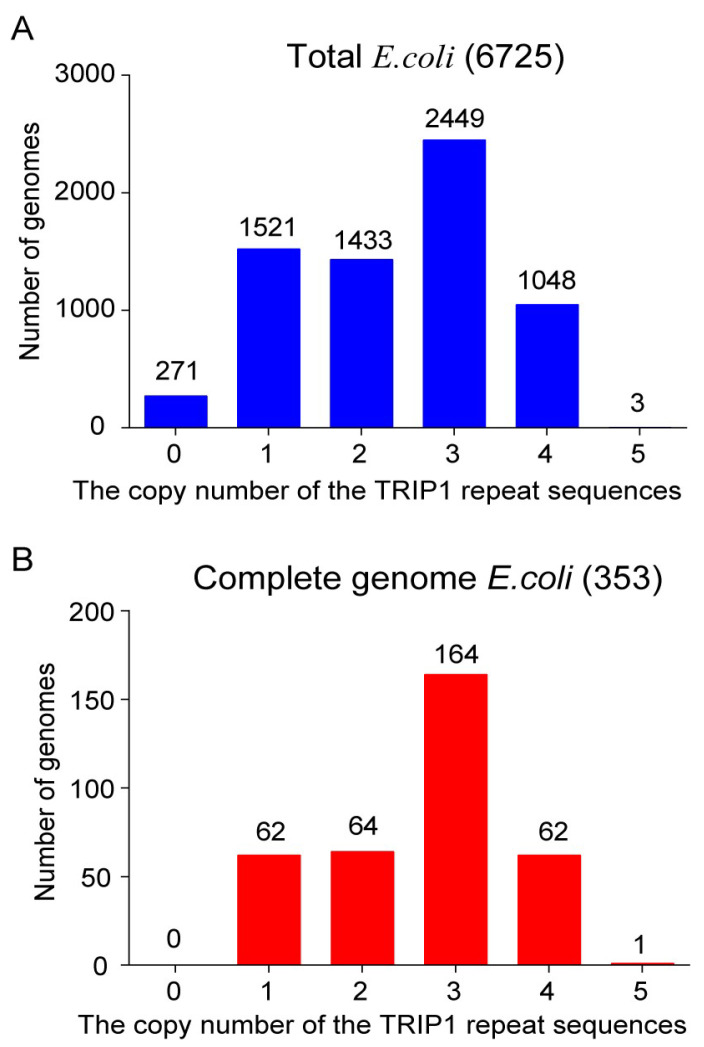
The copy number of the TRIP1 repeat sequences in the *E. coli* genome. The TRIP1 repeat sequences were searched in 6725 available genomes, and the copy per genome was recorded. The distribution of TRIP1 repeat sequence copies is summarized for both total *E. coli* (**A**) and the complete genome of *E. coli* (**B**).

TRIP1 repeat sequences have been found with three copies in *E. coli* K-12 [26]. With the development of high-throughput sequencing technology [30], thousands of *E. coli* genome sequences are now available. Our primary objective is to determine the copy number of TRIP1 in all these strains. Out of the total 6725 genomes analyzed, 6454 (96%) were identified as having the TRIP1 repeat sequences (Figure 1A). The copy number of the TRIP1 repeat sequences found in each genome varied, ranging from zero to five copies. Most genomes had three copies (2449: 36%), which is the same as *E. coli* K-12. Others had one copy (1521: 23%), two copies (1433: 21%), or four copies (1048: 16%). Only three genomes had five TRIP1 repeat sequences in their genomes. Out of the 6725 genomes, only 353 of them had complete genome sequences. TRIP1 repeat sequences were identified in all 353 complete genomes (Figure 1B). The distribution of TRIP1 copy numbers in the complete genomes was similar to that of the total genomes. Most of the complete genomes had three copies (164: 46%), while others had one copy (62: 18%), two copies (64: 18%), or four copies (62: 18%); only one complete genome had five TRIP1 repeat sequences. The copy number of TRIP1 repeat sequences varied in each genome, indicating that they were dynamically created and removed during the genome evolution of the *E. coli* genome.

### 3.2. Distribution and Genome Localization of the Five Types of TRIP1 Repeats in E. coli

**Figure 2:**
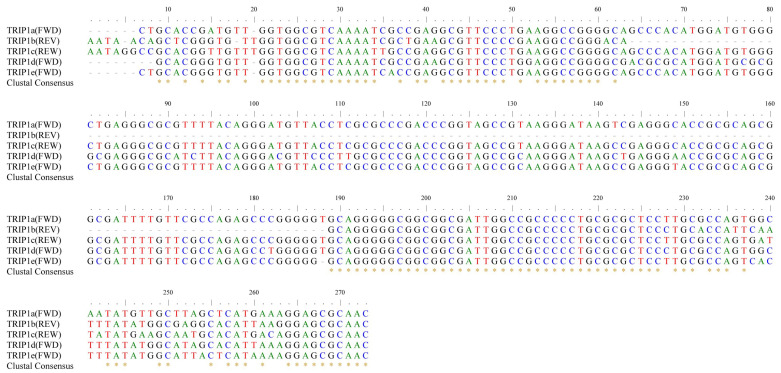
Multiple alignment of the TRIP1 repeats family. The TRIP1a-1e repeat sequences were obtained from NCBI in FASTA format and compared to multiple sequences using BioEdit 7.7 software. The invariant bases are indicated with asterisks. “FWD” stands for the positive direction of gene transcription and “REW” stands for the reverse direction of gene transcription.

Further, we investigated the type and genomic localization of TRIP1 repeats in *E. coli*. The sequences of TRIP1 repeats were compared and analyzed, and the flanking sequences of TRIP1 repeats were extracted and annotated. Finally, we identified five types of TRIP1 repeats in *E. coli*, including three previously reported types (TRIP1a, TRIP1b, and TRIP1c) [26] and two newly discovered types (TRIP1d, and TRIP1e) (Figure 2). All five TRIP1 sequences are GC-rich (62–65% G + C). The TRIP1a repeat sequence is 266 bp in length. TRIP1b is 146 bp in length, which is a partial form of TRIP1a, sharing 84% identity with TRIP1a, and missing an internal 125 bp region. TRIP1c is 273 bp in length and has a 94% identity to TRIP1a. TRIP1d is 264 bp in length and has an 89% identity to TRIP1a. TRIP1e is 265 bp in length and has a 94% identity to TRIP1a. 

**Figure 3:**
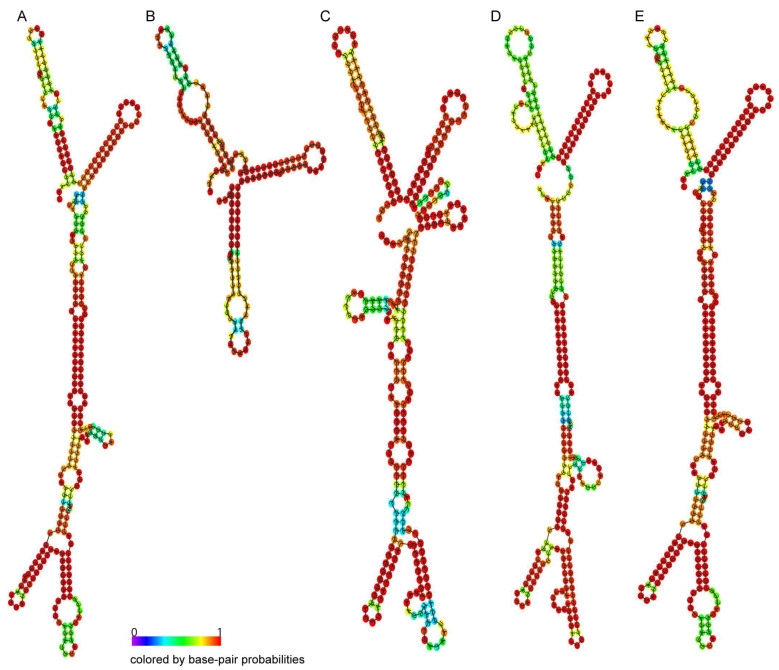
The predicted RNA structure of TRIP1a (**A**), TRIP1b (**B**), TRIP1c (**C**), TRIP1d (**D**), and TRIP1e (**E**). The free energy of the thermodynamic ensemble is predicted to be −143.99 kcal/mol for TRIP1a (**A**), −65.21 kcal/mol for TRIP1b (**B**), −131.36 kcal/mol for TRIP1c (**C**), −145.37 kcal/mol for TRIP1d (**D**), and −149.59 kcal/mol for TRIP1e (**E**). Centroid structure drawings encoding base-pair probabilities of TRIP1a-1e repeats were generated using the RNAfold website: http://rna.tbi.univie.ac.at/cgi-bin/RNAWebSuite/RNAfold.cgi/ accessed on 27 October 2023.

The RNA structure of the five types of TRIP1 repeats was predicted, with higher values indicating greater structural confidence (Figure 3A–E). All five TRIP1 sequences are capable of forming very stable secondary structures in RNA with a conserved double-Y structure. The free energy of the thermodynamic ensemble is predicted to be −143.99 kcal/mol for TRIP1a (Figure 3A), −65.21 kcal/mol for TRIP1b (Figure 3B), −131.36 kcal/mol for TRIP1c (Figure 3C), −145.37 kcal/mol for TRIP1d (Figure 3D), and −149.59 kcal/mol for TRIP1e (Figure 3E). 

**Figure 4:**
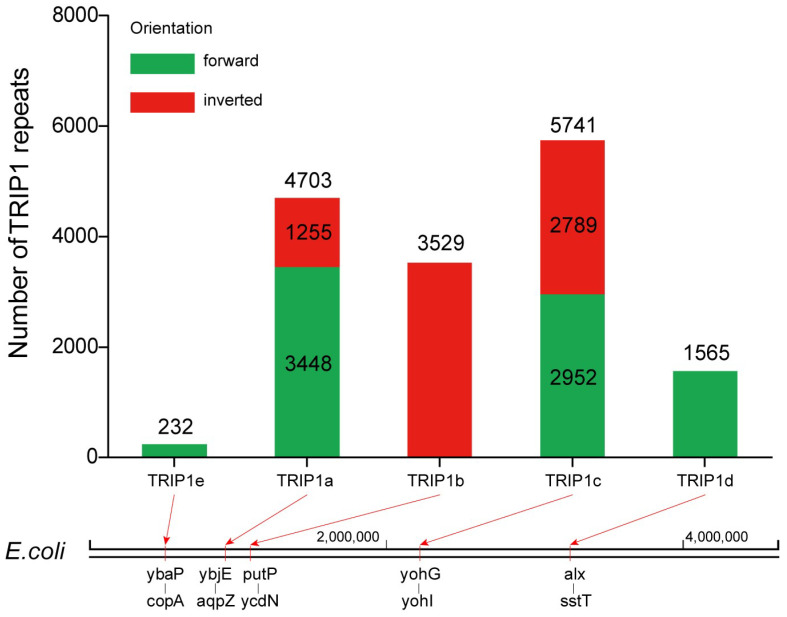
Distribution and genome localization of five types of TRIP1 repeats in *E. coli.* The bottom section demonstrates the position of each type of TRIP1 repeats in the *E. coli* K-12 genome.

The majority of the TRIP1 repeats (15,770/15,941, 99%) were found in five loci. Each type of TRIP1 repeat is localized to a specific chromosomal position (Figure 4). TRIP1a is located 158 bp upstream of the *ybjE* gene start codon and 70 bp downstream of the *aqpZ* gene stop codon. The *ybjE* gene encodes a transmembrane transporter that mediates the export of L-Lysine [31,32]. The *aqpZ* gene encodes a water channel (or aquaporin) that allows bidirectional passive diffusion of water in *E. coli* [33]. TRIP1b is located 41 bp downstream of the *putP* gene stop codon and 355 bp upstream from the *ycdN* gene start codon. The protein encoded by the *putP* gene is a member of the SSS family of sodium/solute transporters [34]. The *ycdN* gene encodes a ferrous iron transmembrane transporter [35,36]. TRIP1c is located 363 bp upstream of the *yohG* gene start codon and 43 bp downstream from the *yohI* gene stop codon. The *yohG* gene encodes an outer membrane efflux protein that associates with multidrug resistance [37]. The *yohI* gene encodes a protein that binds to FMN and has tRNA dihydrouridine synthetase activity [38]. TRIP1d is located 96 bp downstream of the *alx* gene stop codon and 255 bp upstream from the *sstT* gene start codon. The protein encoded by the *alx* gene is a putative membrane-bound redox modulator [39]. The protein encoded by the *sstT* gene is a member of the DAACS family of sodium ion-coupled serine/threonine complexes with neutral L-amino acid transmembrane transporter activity [40]. TRIP1e is located 124 bp upstream of the *ybaP* gene start codon and 38 bp downstream from the *copA* gene stop codon. The *ybaP* gene encodes a TraB family protein whose function is unknown [41]. The *copA*-encoded protein is a member of the P-type ATPase cation transporter family and participates in copper homeostasis primarily as an ATP-dependent monovalent copper exporter under aerobic conditions [42]. These results showed that most of the genes upstream and downstream of the TRIP1 repeat sequences encode transmembrane transporters associated with the pathogenicity and resistance of *E. coli*. 

Moreover, five types of TRIP1 repeats occur at different frequencies in *E. coli* (Figure 4). In the 6725 *E. coli* genomes, TRIP1a is within 4703 genomes, with 3448 in the forward (green) direction and 1255 in the reverse (red) direction. TRIP1b is within 3529 genomes, all of which are in the reverse direction. TRIP1c is within 5741 genomes, with 2952 in the forward direction and 2789 in the reverse direction. TRIP1d is within 1565 genomes, all of which are in the forward direction. TRIP1e is within 232 genomes, all of which are in the forward direction. 

### 3.3. Chromosomal Rearrangements Caused by TRIP1 Repeat Sequences

**Figure 5:**
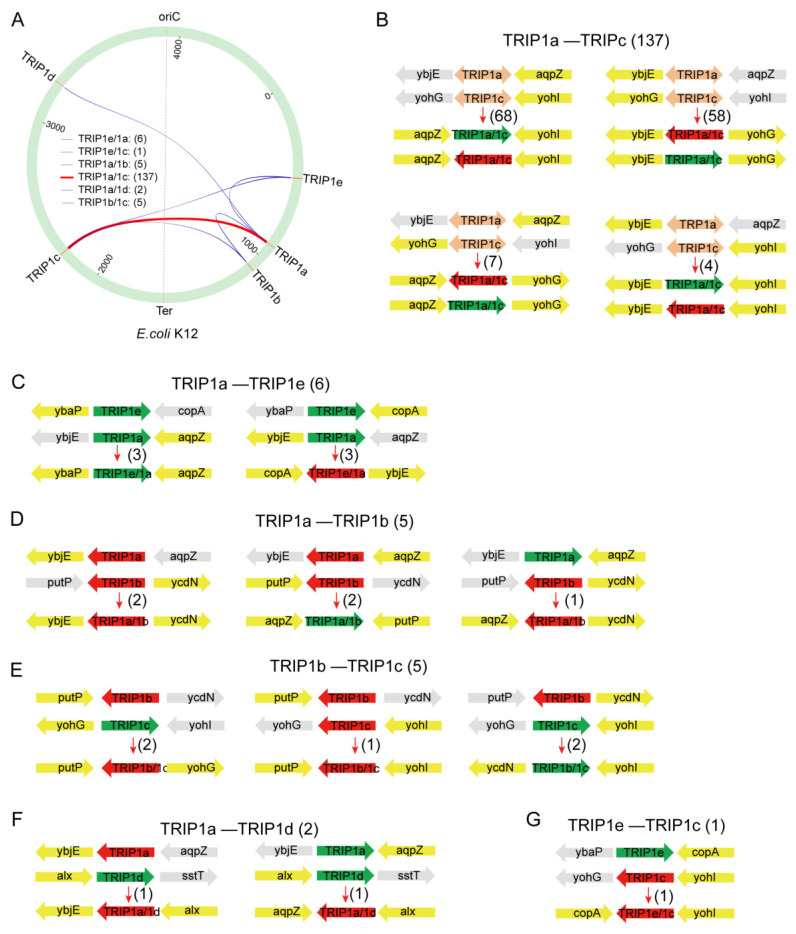
Intra-chromosomal rearrangement caused by the TRIP1 repeat sequence. (**A**) The circle represents the genome of *E. coli* K-12, with the location of each type of TRIP1 repeat indicated. The terms oriC and Ter denote the origin and terminus of chromosome replication, respectively. The line between the two types of TRIP1 repeats represents an intra-chromosomal rearrangement that occurred between them. Six types of rearrangements were identified, with the color and thickness of the line indicating the number of identified rearrangement events. A total of 137 TRIP1a/1c type rearrangement events were found (indicated by the thick red line), while the other five types were much less frequent (indicated by the thin blue line). The number of each rearrangement type is demonstrated. (**B**–**G**) The orientation of the flanking genes for each TRIP1 locus is represented by the direction of the arrow. The direction and color of the arrow represent the orientation of the TRIP1 repeats (green for forward, red for inverted, and orange for both directions). The genes linked together by each rearrangement type are highlighted in yellow. The number of each rearrangement type is indicated in brackets. (**B**) The rearrangements found between TRIP1a and TRIP1c, (**C**) the rearrangements found between TRIP1a and TRIP1e, (**D**) the rearrangements found between TRIP1a and TRIP1b, (**E**) the rearrangements found between TRIP1b and TRIP1c, (**F**) the rearrangements found between TRIP1a and TRIP1d, and (**G**) the rearrangements found between TRIP1e and TRIP1c. Please note that the genes and repeats are not drawn to scale.

In the *E. coli* genomes, a few TRIP1 repeats were found at the junction of flanking genes from two separate loci. This is a typical result of intra-chromosomal rearrangement caused by repeat sequences [6,43,44]. To determine the frequency and influence of chromosomal arrangement caused by TRIP1 repeats, we scanned all genomes for the relative position of the 10 flanking genes from the five loci. We identified a total of 156 rearrangement events in 129 genomes (2%), including six types (Figure 5A). Eighty-eight percent of the rearrangements (137/156) were found between TRIP1a and TRIP1c (Figure 5B), including four rearrangement subtypes. Sixty-eight TRIP1a/1c repeats were between the genes *aqpZ* and *yohI*, fifty-eight TRIP1a/1c repeats were between the genes *ybjE* and *yohG*, seven TRIP1a/1c repeats were between the genes *aqpZ* and *yohG*, and four TRIP1a/1c repeats were between the genes *ybjE* and *yohI*. Moreover, in each rearrangement type, both forward and reverse TRIP1a/1c repeat sequences were also detected. Six rearrangement events were found between TRIP1a and TRIP1e, including three TRIP1a/1e repeats between the genes *ybaP* and *aqpZ* and three TRIP1a/1e repeats between the genes *copA* and *ybjE* (Figure 5C). Five rearrangement events were found between TRIP1a and TRIP1b, including two TRIP1a/1b repeats between the genes *ybjE* and *ycdN*, two TRIP1a/1b repeats between the genes *aqpZ* and *putP*, and one TRIP1a/1b repeat between the genes *aqpZ* and *ycdN* (Figure 5D). Five rearrangement events were found between TRIP1b and TRIP1c, including two TRIP1b/1c repeats between the genes *putP* and *yohG*, one TRIP1b/1c repeat between the genes *putP* and *yohI*, and two TRIP1b/1c repeats between the genes *ycdN* and *yohI* (Figure 5E). Two rearrangement events were found between TRIP1a and TRIP1d, including one TRIP1a/1d repeat between the genes *ybjE* and *alx* and TRIP1a/1d repeat between the genes *aqpZ* and *alx* (Figure 5F). One rearrangement event was found between TRIP1e and TRIP1c, including one TRIP1e/1c repeat between the genes *copA* and *yohI* (Figure 5G). The other four types of rearrangement events, such as TRIP1b/1d, TRIP1b/1e, TRIP1c/1d, TRIP1d/1e, were not identified in the 6725 *E. coli* genomes. In summary, in 6725 *E. coli* genomes, all four possible rearrangement types were found between TRIP1a and other TRIP1 repeats, while only one type of rearrangement event was found between TRIP1d and other TRIP1 repeats.

**Figure 6:**
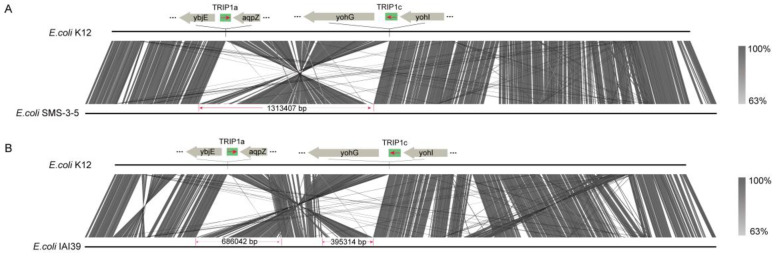
Intra-chromosomal rearrangement caused by TRIP1 repeats in *E. coli* SMS-3-5 and IAI39. (**A**) The alignment between *E. coli* K-12 and *E. coli* SMS-3-5, with the entire region between TRIP1a and TRIP1c being inverted. (**B**) The alignment between *E. coli* K-12 and *E. coli* IAI39, with parts of the intermediate region being inverted. The red arrow marks the orientation of the genes.

Moreover, the rearrangement events were analyzed in two complete genome sequences. In *E. coli* SMS-3-5 [45], the whole genome region between TRIP1a and TRIP1c is inverted compared with *E. coli* K-12 (Figure 6A). The region affected by this rearrangement event is 1,313,407 bp. These rearrangements are likely to have a significant impact on the strain’s phenotype. Similarly, in *E. coli* IAI39 [46], parts of the downstream region of TRIP1a and parts of the upstream region of TRIP1c are inverted, while the middle region between them remains unaffected (Figure 6B). These data showed that the TRIP1 repeats could induce large-scale chromosomal rearrangements in the *E. coli* genomes.

## 4. Discussion

DNA repeats are causes and consequences of genome plasticity [47]. In this study, we systematically analyzed a class of DNA repeat sequences (TRIP1 repeats) that are ubiquitous in the *E. coli* genome but less well-studied. The only recent report on TRIP repeats is that of Rudd et al. from 1999 on their discovery and analysis in *E. coli* K12 [26]. Because recombination and selection for DNA repeats vary between genomes, the number and types of DNA repeats are also quite diverse and in line with ecological variables, such as host-dependent associations or population sizes, and with genetic variables, such as the recombination machinery [47,48]. We found that the copy number of TRIP1 repeat sequences varied across the *E. coli* genome, ranging from zero to five copies, with most of the genomes having three copies (36%, 2449/6725), suggesting a high degree of dynamic change in TRIP1 during *E. coli* evolution. 

We also found that 271 of the 6725 (not completely sequenced) genomes (4%) did not contain any TRIP1 sequences. However, none of the 353 completely sequenced genomes lacked TRIP1. This suggests that most or all of the 4% of the genomes lacking TRIP1 repeat sequences may be due to incomplete sequencing. A comparison of the frequencies of other copies of TRIP1 repeat sequences in completely and incompletely sequenced genomes supports this hypothesis. The proportion of completely sequenced genomes with one to five copies of TRIP1 repeat sequences was always similar to that of incompletely sequenced genomes: 18%/23%, 18%/21%, 46%/36%, 18%/16%, and 0.28%/0.07%, respectively, in contrast to the situation with zero copies.

Five types of TRIP1 repeats, including two novel types (TRIP1d and TRIP1e), were identified in *E. coli* genomes. Five types of TRIP1 repeats occur at different frequencies in *E. coli*, which were located in 70% (4703/6725), 52% (3529/6725), 85% (5741/6725), 23% (1565/6725), and 3% (232/6725) of the genomes, respectively. Moreover, TRIP1 repeats are present in a non-random manner across bacterial genomes, and each type of TRIP1 repeat is localized to a specific locus on the *E. coli* chromosome. 

DNA repeat sequences in bacterial genomes have various functions, including transcribing noncoding RNA [9]. A previous analysis of the RNAseq data available online revealed that out of the 355 DNA repeat elements present in the *E. coli* chromosome, 252 are transcribed [49]. For example, a type of DNA repeat—bacterial Interspersed Mosaic Elements (BIMEs), which are G:C-rich and can form cruciform structures in DNA—can be transcribed into nucleoid-associated non-coding RNA (naRNA) [50]. RNA products of BIMEs are involved in chromosome condensation [9]. Additionally, TRIP1 sequences are also G:C-rich (62–65% G + C) and likely to be capable of forming stable DNA cruciform structures [26]. All five TRIP1 sequences are capable of forming very stable secondary structures in RNA with a conserved double Y structure, suggesting that TRIP1 has the potential to be transcribed into non-coding RNAs with specific physiological functions. 

A bacterial transcription unit consists of several components [51]. In addition to the coding region between the start and stop codons, the core components also include the upstream and downstream non-coding regions. The upstream non-coding region, also known as the promoter region, is tens to hundreds of bps in length. It contains regulatory elements such as promoter sequences and transcription factor binding sites that determine the efficiency and specificity of transcription initiation. The downstream non-coding region is located after the coding region and is a few to tens of bps in length. It may contain termination signals and regulatory elements that affect mRNA processing and stability. In this study, we identified five TRIP1 repeat sequences inserted into the noncoding regions of upstream or downstream gene transcription units. For instance, TRIP1a is located 158 bp upstream of the *ybjE* gene start codon and 70 bp downstream of the *aqpZ* gene stop codon. TRIP1b is located 355 bp upstream from the *ycdN* gene start codon and 41 bp downstream of the *putP* gene stop codon. TRIP1c is located 363 bp upstream of the *yohG* gene start codon and 43 bp downstream from the *yohI* gene stop codon. TRIP1d is located 255 bp upstream from the *sstT* gene start codon and 96 bp downstream of the *alx* gene stop codon. TRIP1e is located 124 bp upstream of the *ybaP* gene start codon and 38 bp downstream from the *copA* gene stop codon. In particular, most of the upstream and downstream genes encode different classes of transmembrane transporters, which are essential for the survival, growth, and adaptation of bacteria in diverse environments. These results suggest that the insertion of these TRIP1 repeat sequences may affect the transcription of these genes, which in turn affects the physiological function of *E. coli*.

DNA repeat sequences could be targeted by recombination processes leading to amplifications, deletions, and rearrangements of genetic material [47,52,53]. In this study, we identified 156 rearrangement events caused by TRIP1 repeat sequences in 6725 *E. coli* genomes (Figure 5). In total, six types of rearrangement events were identified, with the majority (88%, 137/156) occurring between TRIP1a and TRIP1c. Possible reasons for this are the high frequencies of TRIP1a and TRIP1c in the *E. coli* genome, 70% (4703/6725) and 85% (5741/6725), respectively, and their high sequence identity (94%). The region affected by the TRIP1a and TRIP1c rearrangement event can be as long as 1,313,407 bp, as seen in *E. coli* SMS-3-5. Large-scale rearrangements are important in evolution because they can alter chromosome organization and gene expression in ways not possible through point mutations [54]. Thus, we concluded that the large-scale rearrangements between TRIP1 repeats should have a strong influence on the phenotype of these strains, which requires further investigation.

## 5. Conclusions

In summary, we systematically analyzed the diversity, distribution, and chromosomal rearrangements of the TRIP1 repeat sequences in the *E. coli* genome. After screening 6725 *E. coli* genomes, TRIP1 was found to be ubiquitous (96%: 6454/6725) and with many copies in the *E. coli* strains. Five types of TRIP1 repeat sequences were identified in the *E. coli* genomes, and they occurred with varying frequencies in *E. coli*, with TRIP1a and TRIPc occurring at the highest frequencies. More importantly, the TRIP1 repeat sequence can induce chromosomal rearrangement in the *E. coli* genome. A total of 156 rearrangement events were identified, of which 88% (137/156) were between TRIP1a and TRIP1c. In addition, large-scale rearrangements were also identified between TRIP1a and TRIP1c in *E. coli* genomes, such as strain SMS-3-5. All these findings have important implications for future research on TRIP1 repeats.

## Data Availability

All data presented are available in the manuscript.
